# RE: “INVITED COMMENTARY: THE DISILLUSIONMENT OF DEVELOPMENTAL ORIGINS OF HEALTH AND DISEASE (DOHAD) EPIDEMIOLOGY”

**DOI:** 10.1093/aje/kwaa100

**Published:** 2020-06-22

**Authors:** Hazel Inskip, Keith Godfrey, Cyrus Cooper, Mark Hanson, Caroline Fall, Janis Baird, Mary Barker, Deborah Sloboda, Lucilla Poston

We were surprised by the first article in the January issue of the *American Journal of Epidemiology*. With the title “Invited Commentary: The Disillusionment of Developmental Origins of Health and Disease (DOHaD) Epidemiology” (1), we anticipated a broad discussion of epidemiology in DOHaD and a critique of the concept. Instead, we read a commentary on a 2018 paper by Masarwa et al. (2). The commentary notes that this systematic review was not very informative.

We do not understand how criticism of an article on a specific topic could justify a title indicative of disillusionment with the epidemiology of DOHaD in its entirety, especially as the DOHaD concept focuses on developmental exposures *other* than teratology (3). The authors of the commentary themselves note that their criticism of the DOHaD concept is confined to medication use during pregnancy, but their concluding paragraph, which is dismissive of DOHaD, bears no relevance to their opinion of the article. The commentary’s title, while possibly reflecting the opinion of the authors, misrepresents the contents of the commentary, and we ask that it be amended and an erratum published.
